# Supplementary material for: Targeting an allosteric site in dynamin-related protein 1 to inhibit Fis1-mediated mitochondrial dysfunction
Source: Nat Commun. 2023 Jul 19;14:4356. doi: 10.1038/s41467-023-40043-0 (PMC10356917; doi:10.1038/s41467-023-40043-0)
Supplement: Supplementary file 1 — Supplementary Information [file 41467_2023_40043_MOESM1_ESM.pdf]

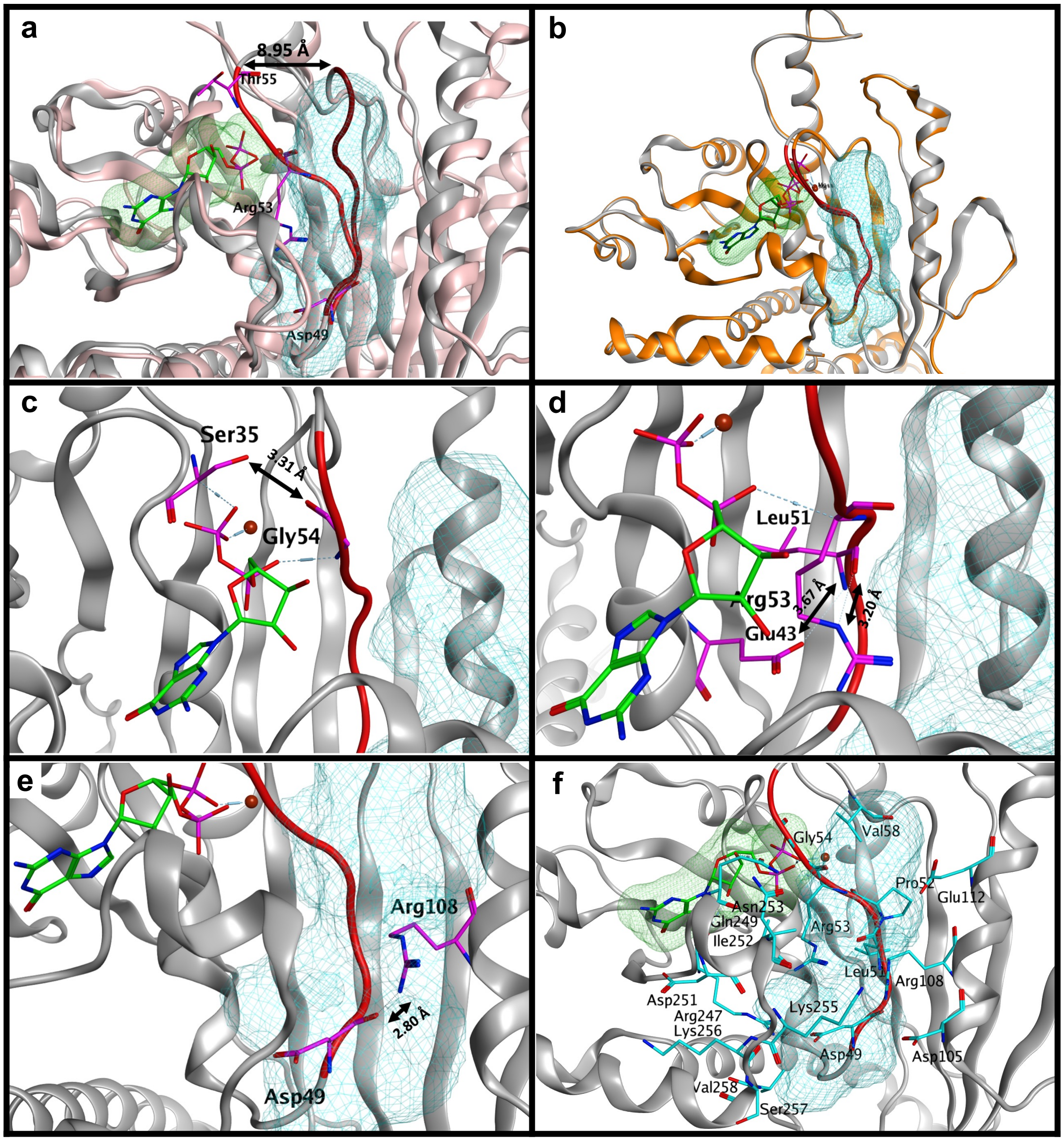

Supplementary Fig. 1 | Drp1 switch I interactions and switch I adjacent groove (SWAG). a, Overlay of the APO structure 4BEJ peach) and the GDP.AlF4 bound structure 3W6P (grey) with side chains of P110 residues D49, R53, and T55 labeled red and GDP.AlF4 labeled green. The black arrow shows switch I's 8.95 angstrom swing toward the GTP binding site upon nucleotide binding. **b,** Overlay of the GDP.AlF4 bound structure 3W6P (grey) and GMPPCP bound structure 3W6O (orange). **c,** Interaction between S35 (P-loop) and G54 (switch I/P110). **d**, Switch I tethering interaction between R53 side chain, L51 backbone, and D43 side chain. **e,** Switch I tethering interaction between D49 backbone and R108 side chain. **f**, SWAG residues labeled (cyan).

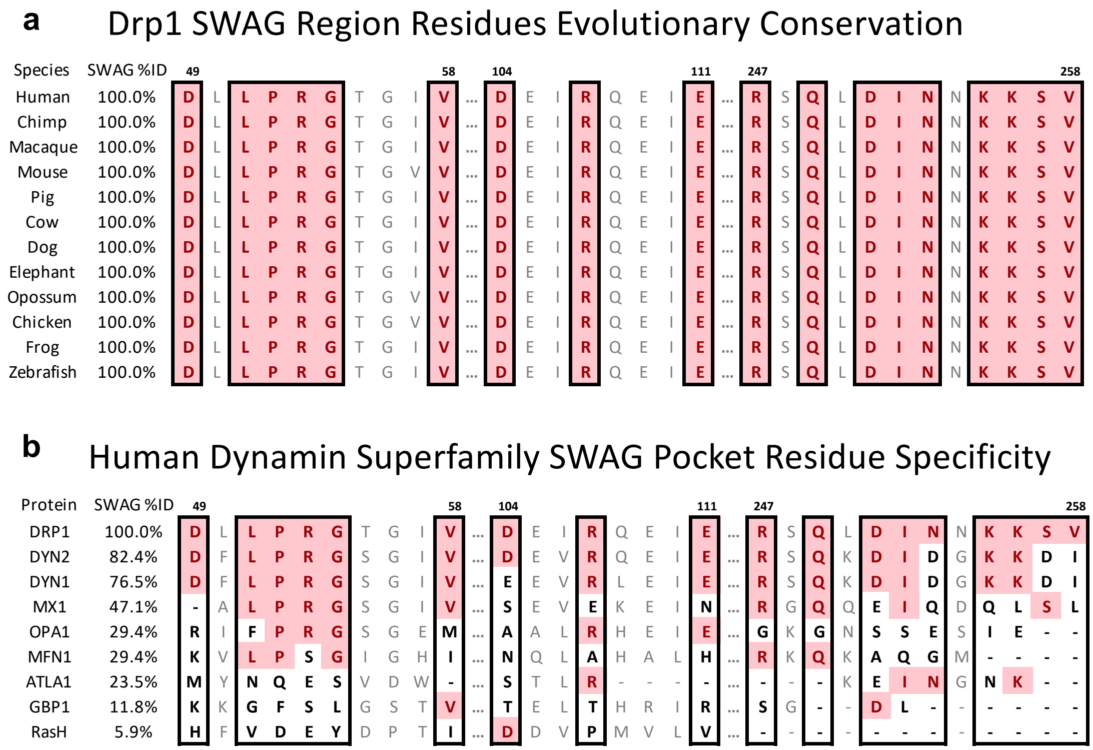

Supplementary Fig. 2 | Drp1 evolutionary conservation and comparison to other human GTPases. a, Drp1 evolutionary conservation of the SWAG residues from human to zebrafish (grey residues outside of box are not part of the SWAG). **b,** Multiple sequence alignment of Drp1 with related human GTPases. Red residues are identical to the Drp1 SWAG and the percentage of identical residues is indicated.

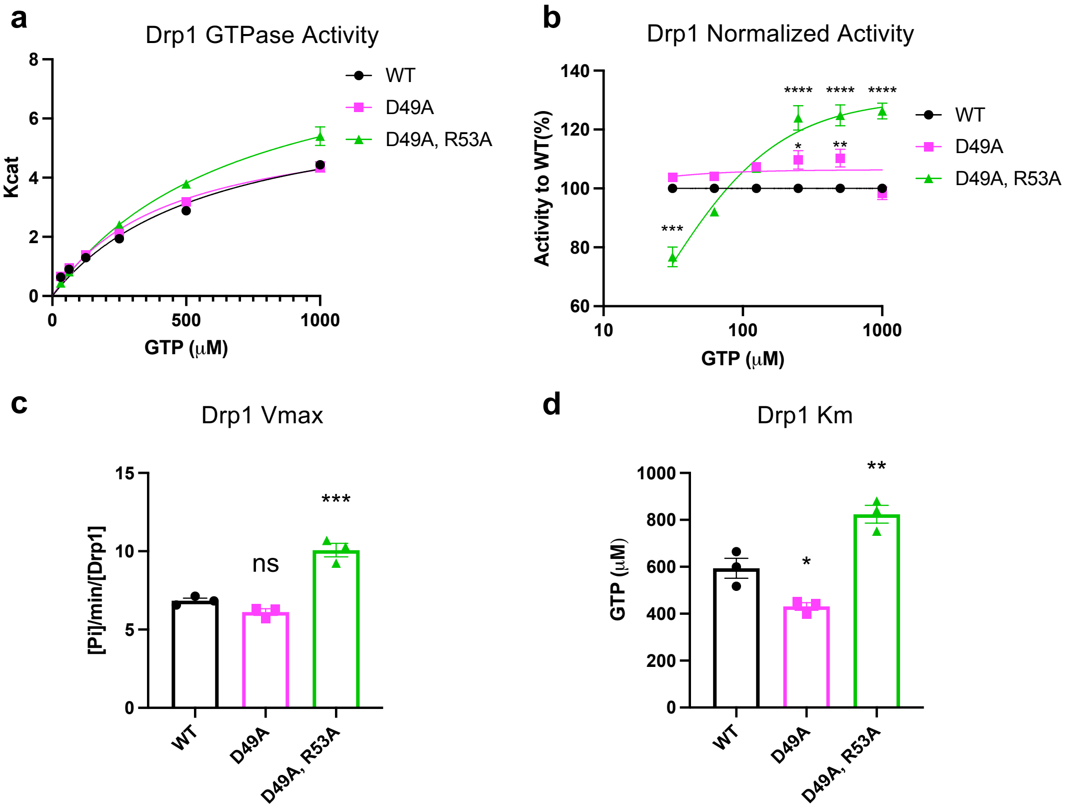

Supplementary Fig. 3 | GTPase activity of Drp1 mutants. a-d, GTPase kinetics of recombinant WT, [D49A], and [D49A, R53A] Drp1 (3 experiments with 3 replicates). Significance determined by one-way ANOVA with Dunnett's multiple comparisons test (each group against WT, two-tailed test). Error bars show mean with SEM. The p-value is indicated by stars (ns = $p > .05$, * = $p < .05$, ** = $p < .01$, *** = $p < .001$, and **** = $p < .0001$). Source data including all statistics (degrees of freedom, p values, effect sizes, and confidence intervals) are provided in the Source Data file.

Supplementary Table 1. Predicted physiochemical properties

Serial Number	Mass (Da)	S	Fraction Csp3	#Rotatable bonds	#H-bond acceptors	#H-bond donors	TPSA	WLOGP	Solubility (mg/ml)	GI absorption	Pgp substrate
SC1	464.6	-8.38	0.65	14	4	6	109.6	-2.29	3.33	Low	Yes
SC3	479.9	-8.09	0.22	10	7	2	135.69	2.41	0.0283	High	Yes
SC9	466.5	-7.85	0.24	7	6	2	169.57	0.98	0.119	Low	Yes

Supplementary Table 2. Predicted physiochemical properties (cont.)

Serial Number	CYP1A2 inhibitor	CYP2C19 inhibitor	CYP2C9 inhibitor	CYP2D6 inhibitor	CYP3A4 inhibitor	Lipinski #violations	Ghose #violations	Veber #violations	Egan #violations	Muegge #violations
SC1	No	No	No	No	Yes	1	3	1	0	1
SC3	No	Yes	Yes	No	Yes	0	1	0	1	0
SC9	No	No	No	No	Yes	1	0	1	1	1

Supplementary Tables | Swiss ADME predicted physiochemical properties. S is the predicted binding affinity for Drp1 generated in M.O.E. using in silico docking. The rest of the data were exported from the SwissADME online prediction tool (www.swissadme.ch). Fraction Csp3 is the ratio of sp3 hybridized carbons over the total carbon count of the molecule. TSPA is total polar surface area. WLOGP is an estimate of the partition coefficient (lipophilicity). Solubility (in water) is estimated using the ESOL method. The methods for estimating these values are described in Daina 2017 (doi.org/10.1038/srep42717).

Compound Table

Label	Tgt Score	Mass Error (ppm)	Tgt Formula	Obs. RT	Ref. Mass	Obs. Mass
Cpd 3: C23 H38 N6 O4; 0.473	99.38	-0.77	C23 H38 N6 O4	0.473	462.2955	462.2951

Obs. *m/z*	Obs. RT	Obs. Mass	Tgt Formula	Tgt Mass	Tgt Mass Error (ppm)	RT Diff.	Find Cpds Algorithm
463.3025	0.473	462.2951	C23 H38 N6 O4	462.2955	-0.77	Find By Formula	

Compound Chromatograms

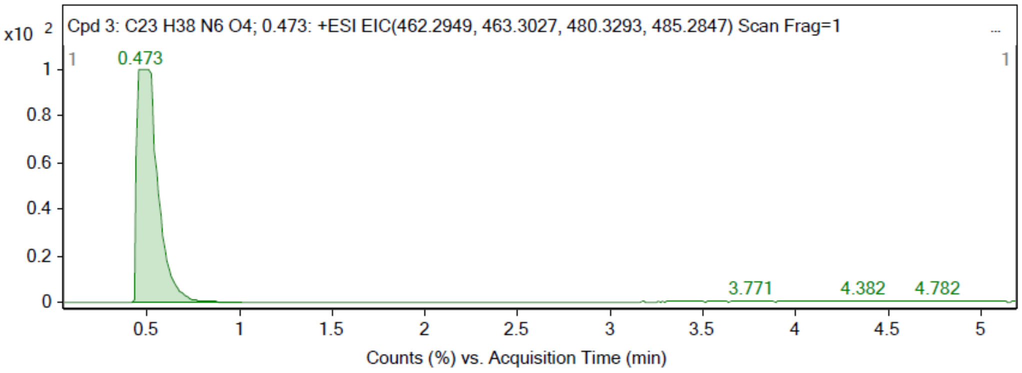

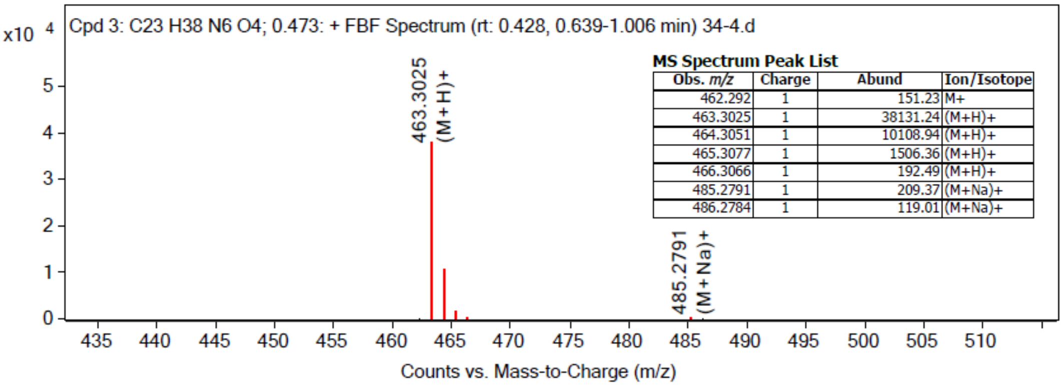

Obs. *m/z*	Charge	Abund	Ion/Isotope
462.292	1	151.23	M+
463.3025	1	38131.24	(M+H)+
464.3051	1	10108.94	(M+H)+
465.3077	1	1506.36	(M+H)+
466.3066	1	192.49	(M+H)+
485.2791	1	209.37	(M+Na)+
486.2784	1	119.01	(M+Na)+

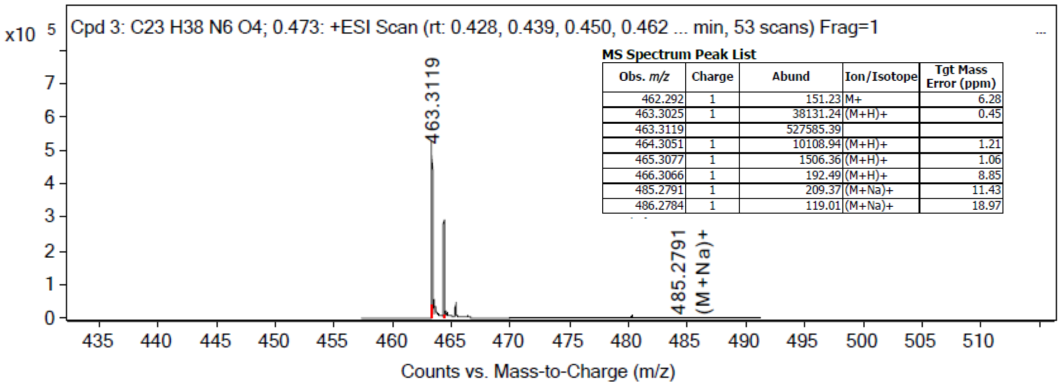

Obs. *m/z*	Charge	Abund	Ion/Isotope	Tgt Mass Error (ppm)
462.292	1	151.23	M+	6.28
463.3025	1	38131.24	(M+H)+	0.45
463.3119		527585.39		
464.3051	1	10108.94	(M+H)+	1.21
465.3077	1	1506.36	(M+H)+	1.06
466.3066	1	192.49	(M+H)+	8.85
485.2791	1	209.37	(M+Na)+	11.43
486.2784	1	119.01	(M+Na)+	18.97

Supplementary Fig. 4a | SC1 HRMS quality control data

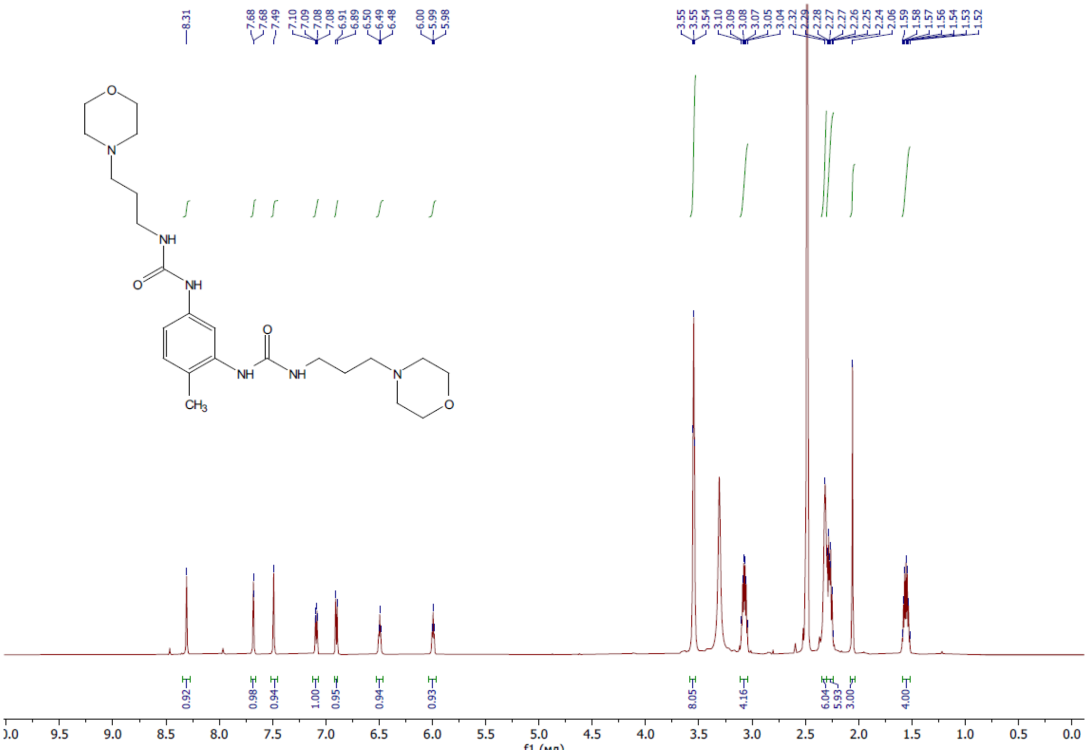

Supplementary Fig. 4b | ^{1}H NMR spectrum of **SC1**

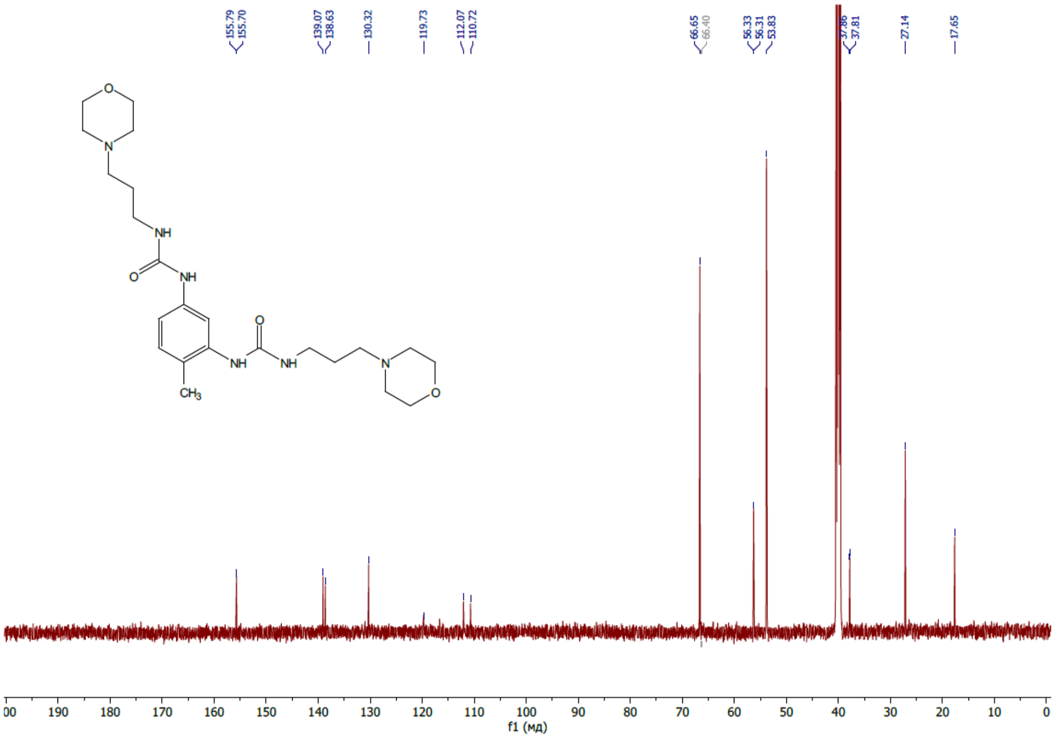

Supplementary Fig. 4c | ^{13}C NMR spectrum of **SC1**.

Compound Table

Label	Tgt Score	Mass Error (ppm)	Tgt Formula	Obs. RT	Ref. Mass	Obs. Mass
Cpd 1: C23 H22 Cl N7 O3; 4.128	97.84	-1.74	C23 H22 Cl N7 O3	4.128	479.1473	479.1464

Obs. *m/z*	Obs. RT	Obs. Mass	Tgt Formula	Tgt Mass	Tgt Mass Error (ppm)	RT Diff.	Find Cpds Algorithm
480.1535	4.128	479.1464	C23 H22 Cl N7 O3	479.1473	-1.74	Find By Formula	

Compound Chromatograms

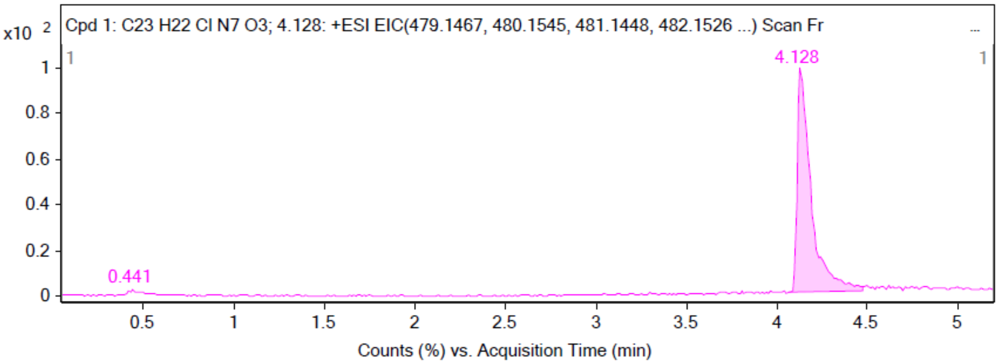

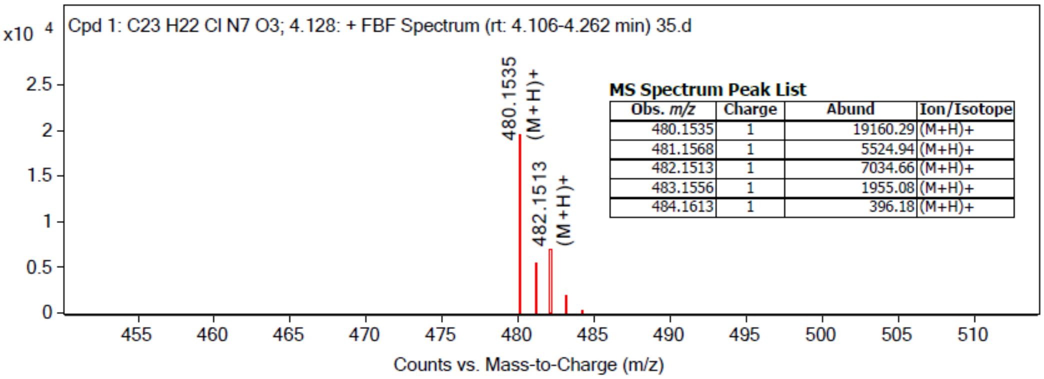

MS Spectrum Peak List

Obs. *m/z*	Charge	Abund	Ion/Isotope
480.1535	1	19160.29	(M+H)+
481.1568	1	5524.94	(M+H)+
482.1513	1	7034.66	(M+H)+
483.1556	1	1955.08	(M+H)+
484.1613	1	396.18	(M+H)+

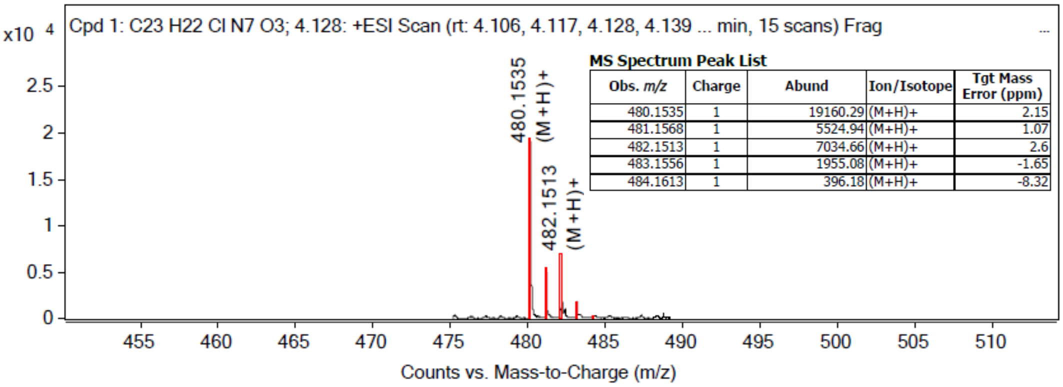

MS Spectrum Peak List

Obs. *m/z*	Charge	Abund	Ion/Isotope	Tgt Mass Error (ppm)
480.1535	1	19160.29	(M+H)+	2.15
481.1568	1	5524.94	(M+H)+	1.07
482.1513	1	7034.66	(M+H)+	2.6
483.1556	1	1955.08	(M+H)+	-1.65
484.1613	1	396.18	(M+H)+	-8.32

Supplementary Fig. 5a | SC3 HRMS quality control data

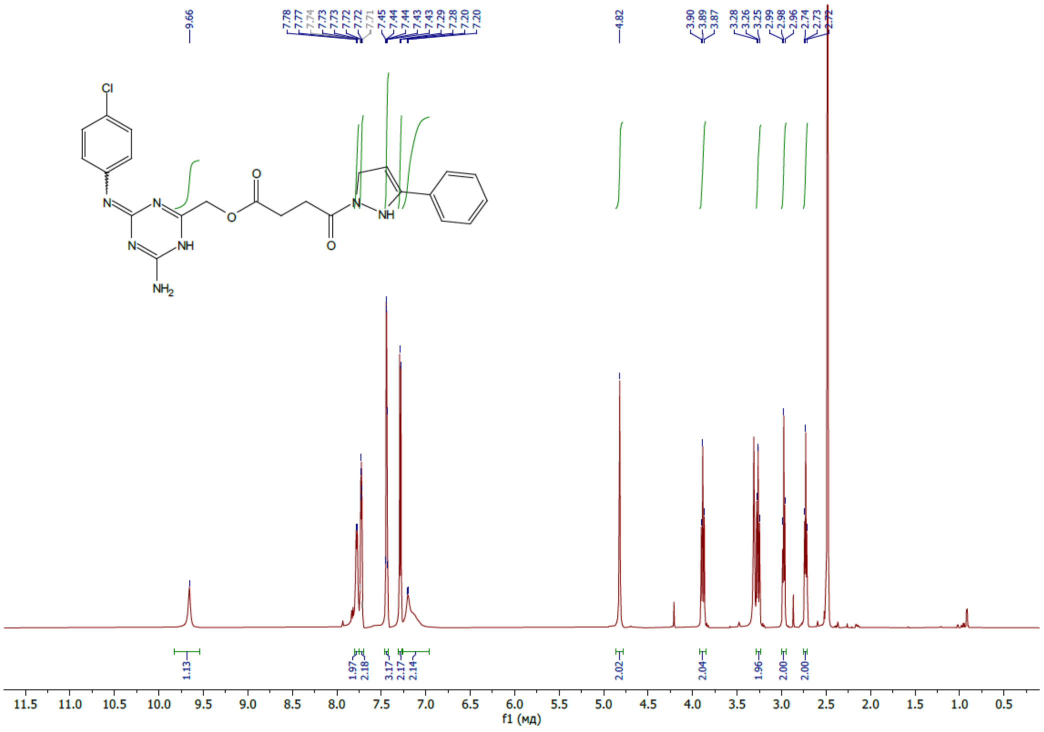

Supplementary Fig. 5b | ^{1}H NMR spectrum of **SC3**.

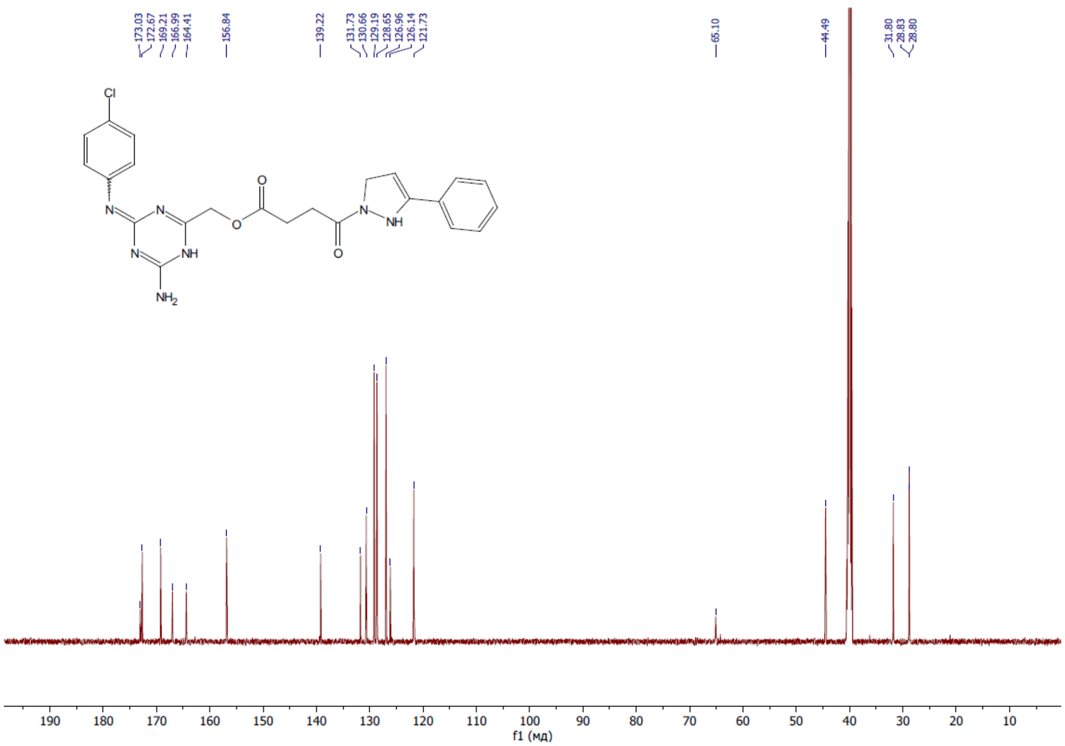

Supplementary Fig. 5c | ^{13}C NMR spectrum of **SC3**.

Compound Table

Label	Tgt Score	Mass Error (ppm)	Tgt Formula	Obs. RT	Ref. Mass	Obs. Mass
Cpd 2: C21 H22 N8 O3 S; 0.535	97.74	-1.66	C21 H22 N8 O3 S	0.535	466.1536	466.1528

Obs. *m/z*	Obs. RT	Obs. Mass	Tgt Formula	Tgt Mass	Tgt Mass Error (ppm)	RT Diff.	Find Cpds Algorithm
467.1602	0.535	466.1528	C21 H22 N8 O3 S	466.1536	-1.66	Find By Formula	

Compound Chromatograms

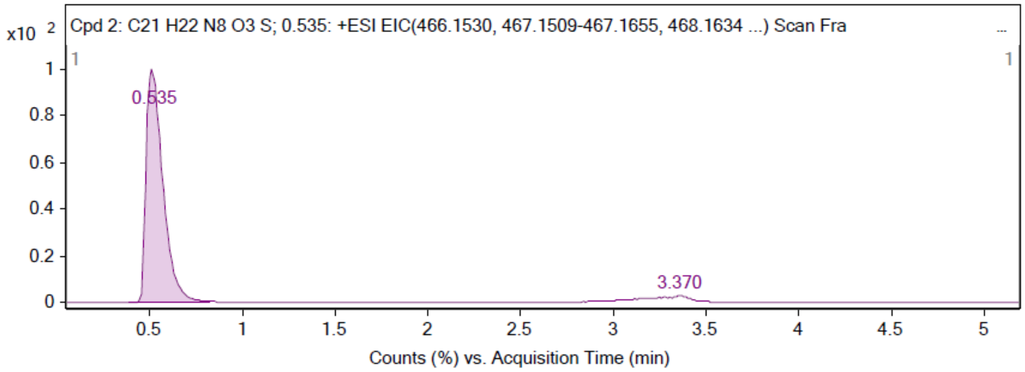

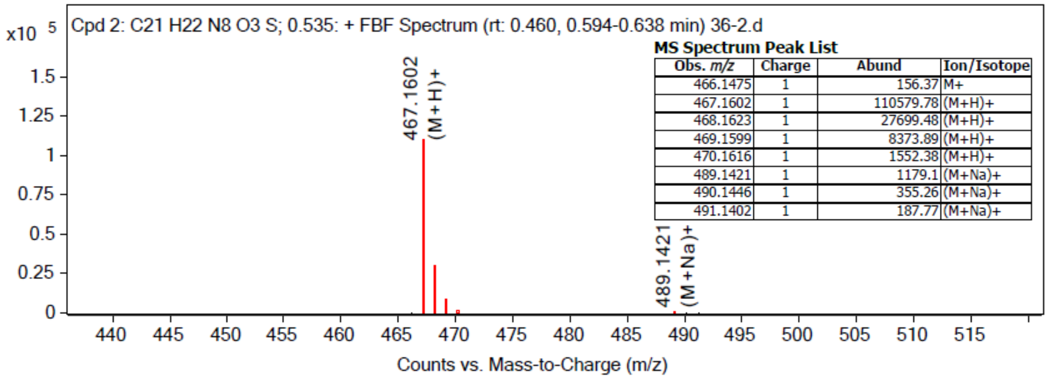

MS Spectrum Peak List

Obs. *m/z*	Charge	Abund	Ion/Isotope
466.1475	1	156.37	M+
467.1602	1	110579.78	(M+H)+
468.1623	1	27699.48	(M+H)+
469.1599	1	8373.89	(M+H)+
470.1616	1	1552.38	(M+H)+
489.1421	1	1179.1	(M+Na)+
490.1446	1	355.26	(M+Na)+
491.1402	1	187.77	(M+Na)+

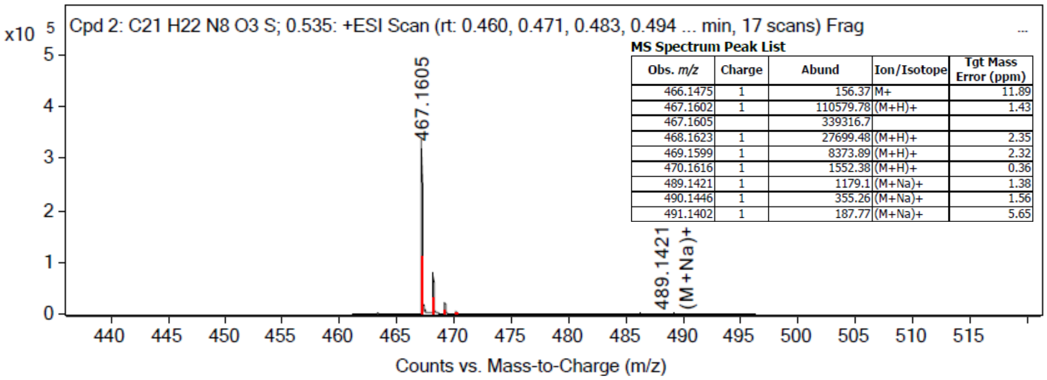

MS Spectrum Peak List

Obs. *m/z*	Charge	Abund	Ion/Isotope	Tgt Mass Error (ppm)
466.1475	1	156.37	M+	11.89
467.1602	1	110579.78	(M+H)+	1.43
467.1605		339316.7		
468.1623	1	27699.48	(M+H)+	2.35
469.1599	1	8373.89	(M+H)+	2.32
470.1616	1	1552.38	(M+H)+	0.36
489.1421	1	1179.1	(M+Na)+	1.38
490.1446	1	355.26	(M+Na)+	1.56
491.1402	1	187.77	(M+Na)+	5.65

Supplementary Fig. 6a | SC9 HRMS quality control data.

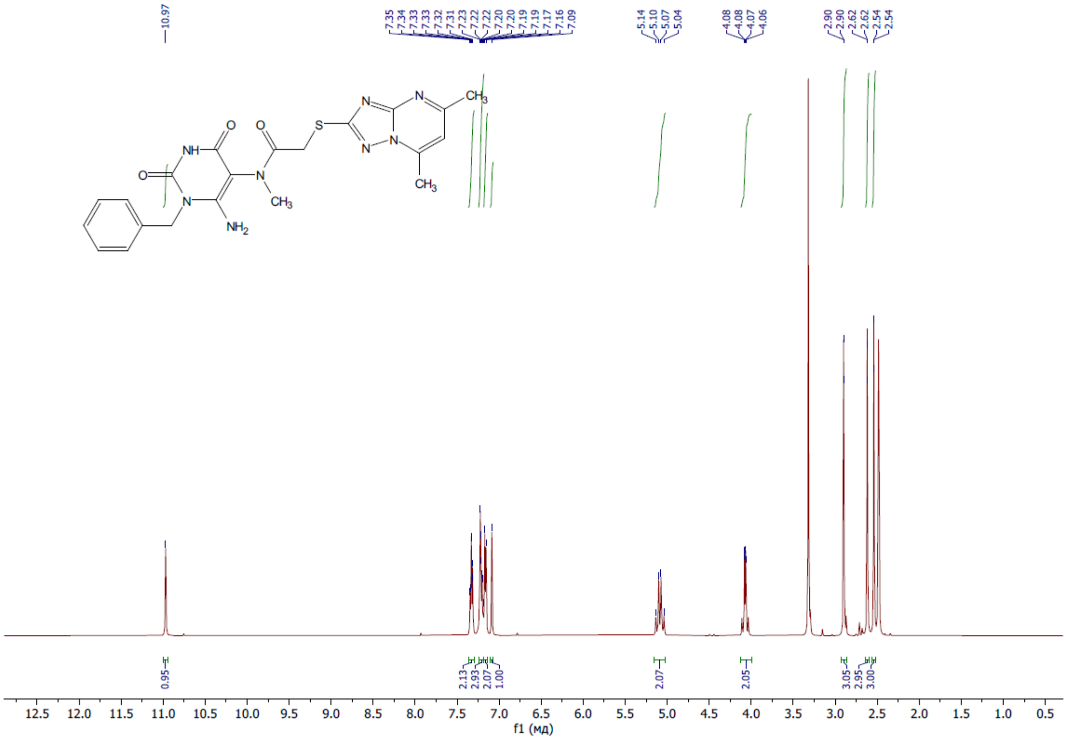

Supplementary Fig. 6b | ^{1}H NMR spectrum of **SC9**.

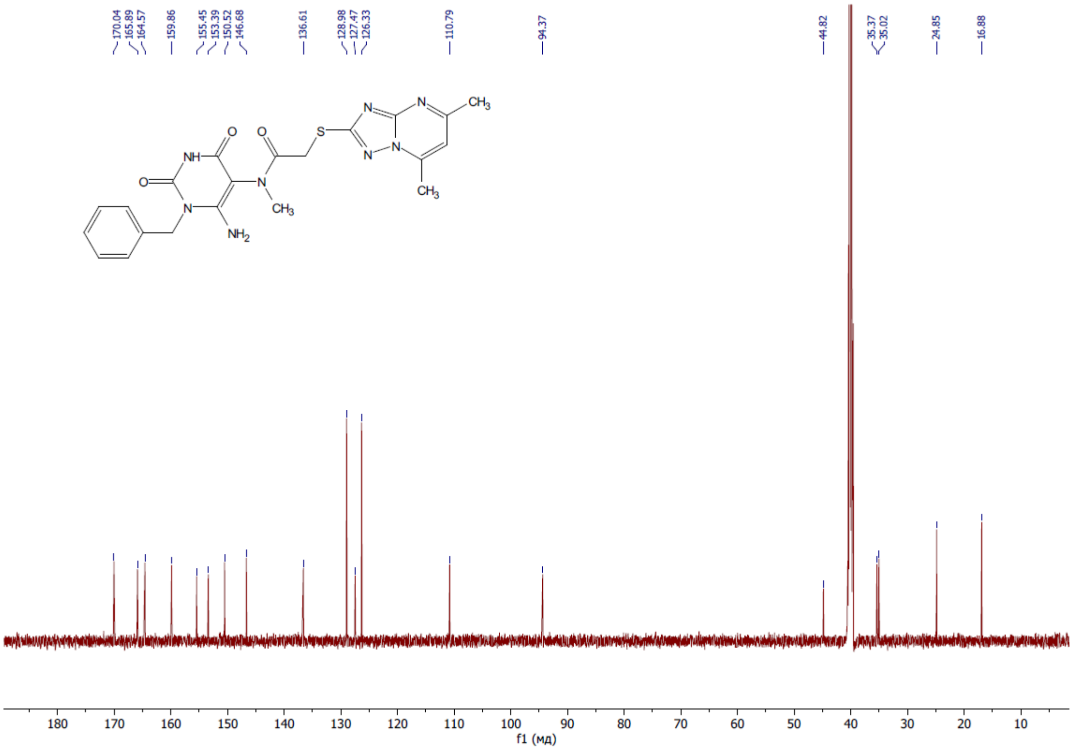

Supplementary Fig. 6c | ^{13}C NMR spectrum of **SC9**.

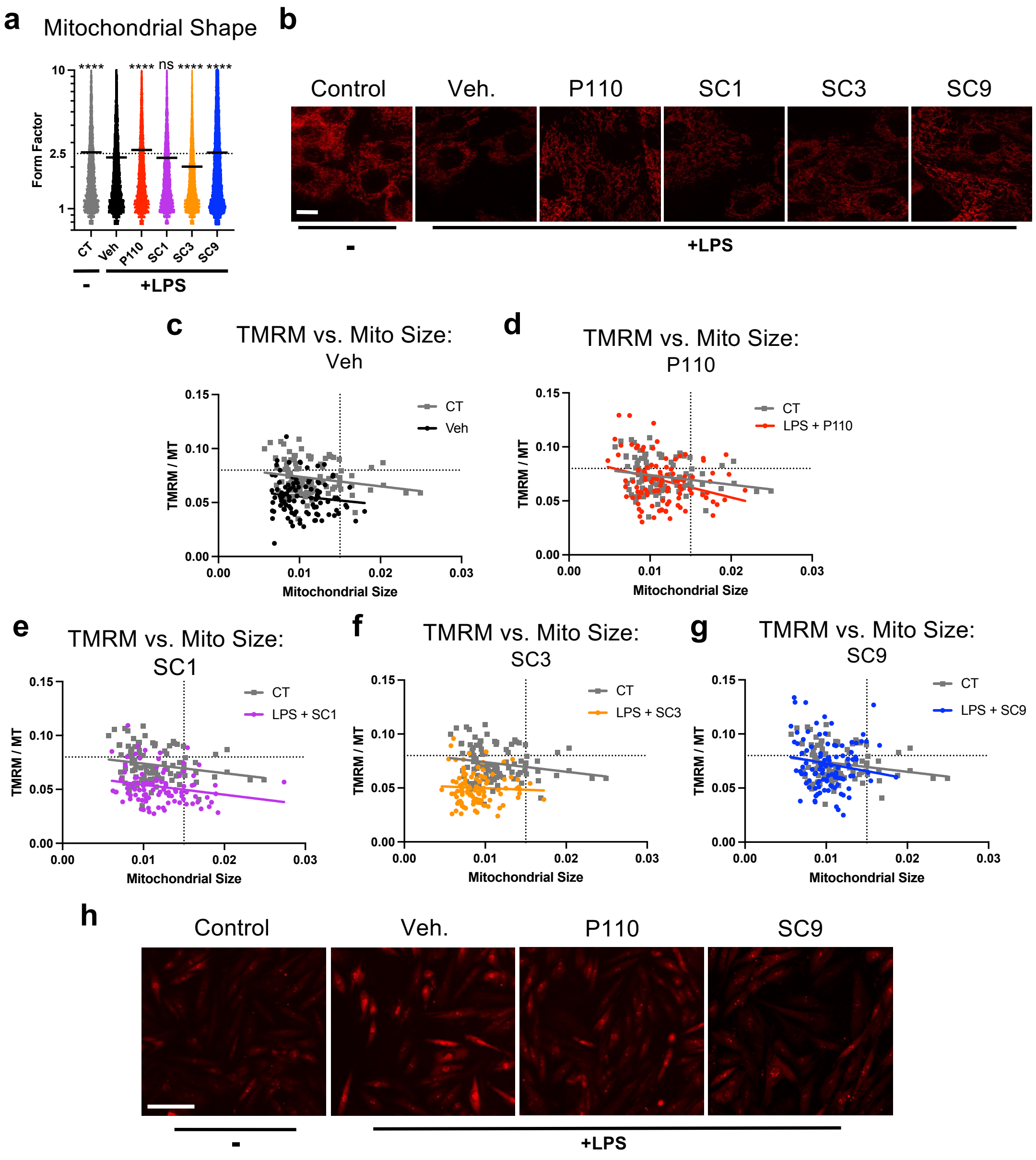

Supplementary Fig. 7| Single cell mitochondrial size and membrane potential. a, Form factor calculated from pooled mitochondria from all analyzed cells after LPS (2ug/ml) and 2μM compound co-treatment for 16hrs (CT: n=9443 mitochondria; LPS: n=15,238 mitochondria; P110: n=15,396 mitochondria; SC1: n=15,181 mitochondria; SC3; n=15,943 mitochondria; SC9: n=12,188 mitochondria; data pooled from 2 experiments). **b,** Representative 60x images of H9c2 cells treated with LPS and stained with TMRM after 16hrs (white scale bar = 10μm). **c-g** H9c2 cells co-stained with TMRM and MitoTracker Deep Red FM to estimate both mitochondrial membrane potential (TMRM integrated density/MitoTracker 2D area) and mitochondrial size (n=103 cells per condition; data are pooled from 2 experiments). Single cell manual segmentation was conducted by an observer blinded to the experimental conditions. **h,** Representative 10x images of H9c2 cells treated with LPS and stained with MitoSOX after a 24hr incubation (white scale bar = 100μm). Error bars in (**a**) show mean with SEM (though very small) and one-way ANOVA with Dunnett's multiple comparisons test (each group against Veh., two-tailed test). P-value results indicated by stars (ns = p > .05, * = p < .05, ** = p < .01, *** = p< .001, and **** = p < .0001). Non-significant p-values < .1 are written above bar. Source data including all statistics (degrees of freedom, p values, effect sizes, and confidence intervals) are provided in the Source Data file.

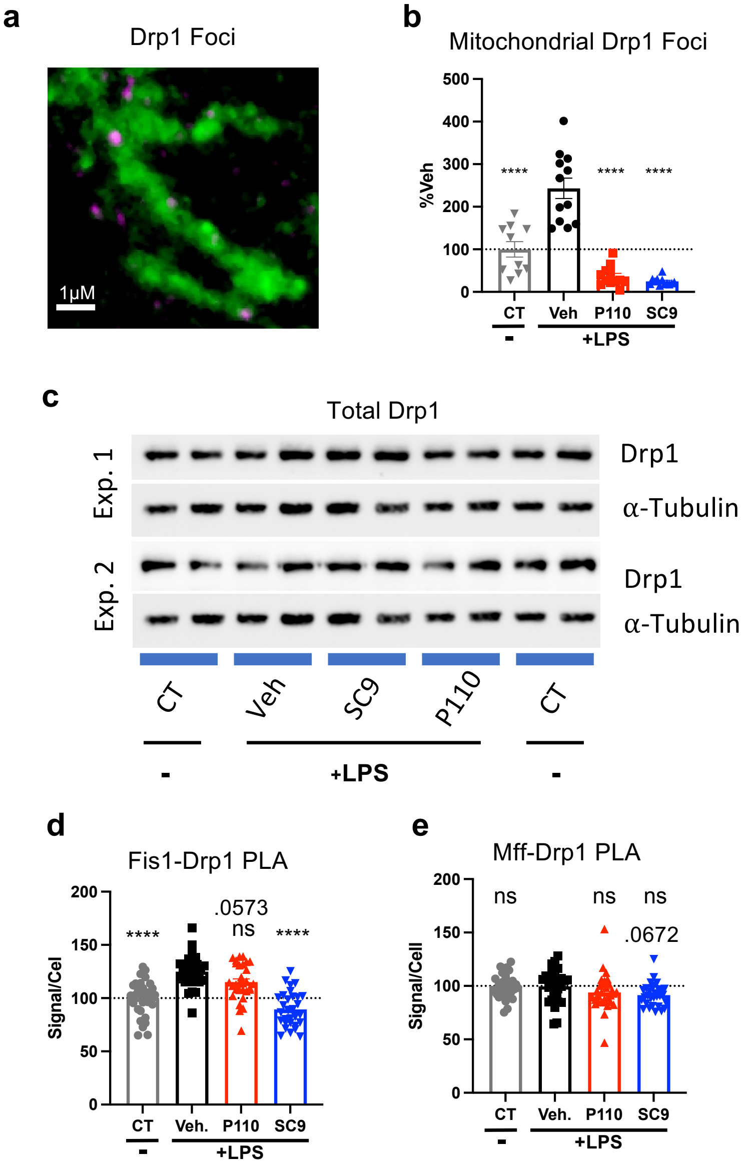

Supplementary Fig. 8| Drp1 Foci, Total Drp1, and PLA analysis (non-single cell). a, Drp1 foci (magenta) and mitochondria (Fis1) immuno-staining result imaged at 100x with a single focal-plane. The image shows the colocalization of Drp1 foci with mitochondria**. b,** Quantification of total mitochondria-associated Drp1 signal (integrated density) per mitochondrial area with each data point representing one image (CT: n=10; Veh: n=12; P110: n=12; SC9: n=12; pooled data from two experiments. Z-stacks taken at 100x magnification. **c,** Western blots (quantified in Figure. 5c) results showing the total levels of Drp1 after LPS and compound treatment (CT: n=8; Veh: n=4; P110: n=4; SC9: n=4). Drp1-Fis1 **(d)** and Drp1-Mff **(e)** PLA signal per cell after LPS treatment (data from 2 experiments pooled with 30 images quantified and plotted per condition). Error bars show mean with SEM and one-way ANOVA with Dunnett's multiple comparisons test (each group against Veh., two-tailed test). P-value results indicated by stars (ns = p > .05 and **** = p < .0001). Non-significant p-values < .1 are written above bar. Source data including all statistics (degrees of freedom, p values, effect sizes, and confidence intervals) are provided in the Source Data file.

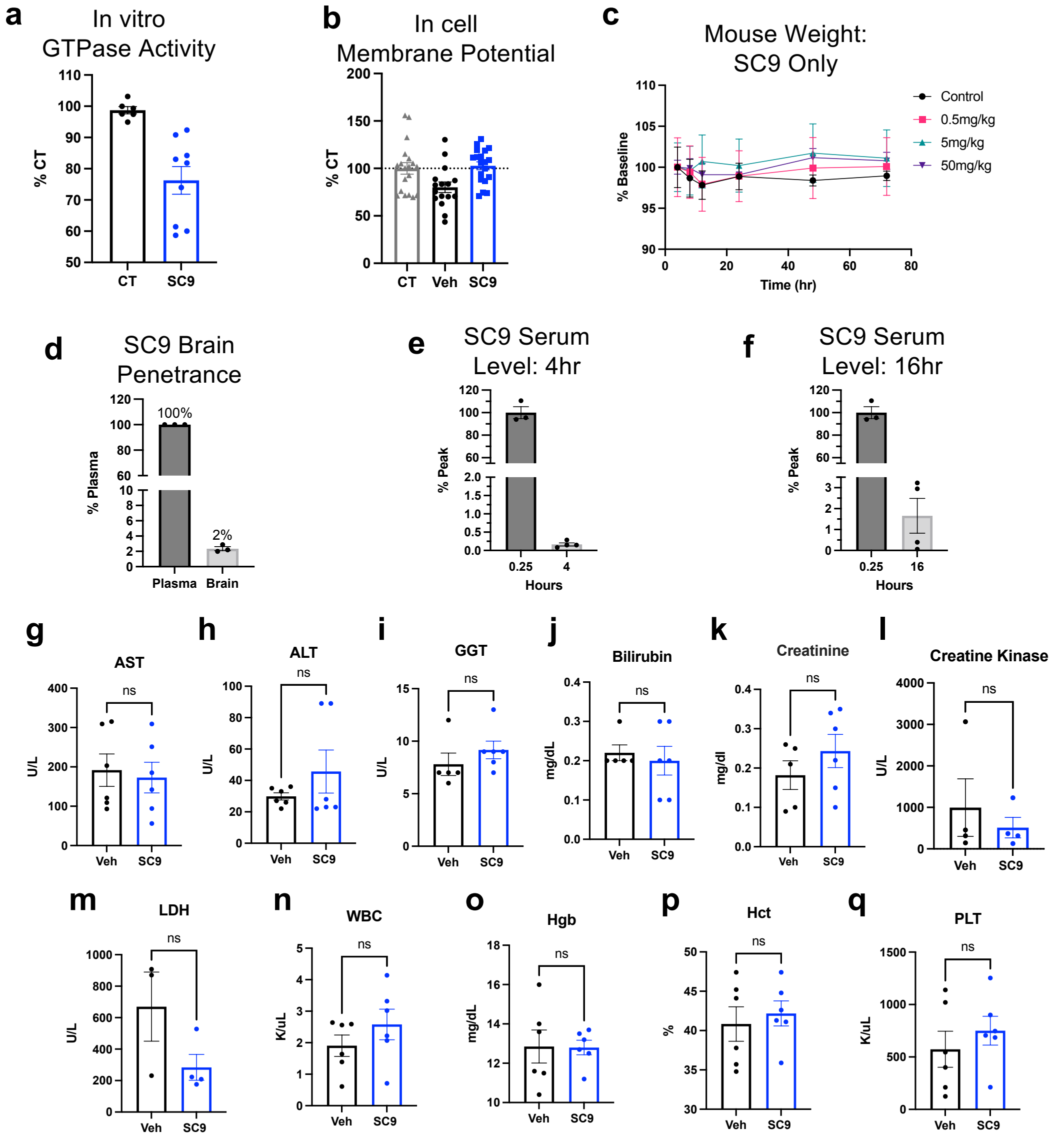

Supplementary Fig. 9| a, Inhibition of Drp1 GTPase activity *in vitro* (CT: n=6; SC9: n=9; data from 3 experiments with 3 replicates each). **b,** Data from Fig. 6b on inhibiting LPS-induced mitochondrial membrane depolarization with 10μM SC9 (CT: n=19; Veh: n=16; SC9 n=19; data from 4 experiments pooled). **c,** Mice injected I.P. with an increasing dose of SC9 and observed for signs of toxicity for 72hrs by recording body mass and periodically scoring with the murine sepsis score (n=4 mice for CT, 0.5mg, and 5mg and n=3 mice for 50mg; 1 experiment). **d,** Levels of SC9 plasma to brain ratio measured with mass spectrometry, sampled 15 minutes after I.P. injection of SC9 (20mg/kg; 3 mice per condition). **e,** Plasma concentration of SC9 measured with mass spectrometry, sampled at 15 minutes and 4 hours after co-administration of 20mg/kg SC9 and 0.3mg LPS (3 mice at 15min and 4 mice at 4 hours). **f,** Plasma concentration of SC9 measured with mass spectrometry, sampled at 15 minutes and 16 hours after co-administration of 10mg/kg SC9 and 0.3mg LPS. Mice re-dosed with additional 10mg/kg at 4 hours. (3 mice at 15min and 4 mice at 16 hours). **g-q**, Animal toxicology serum measurements in mice dosed with 50mg/kg SC9 or vehicle control (DMSO) at 72hrs post injection. **g-j,** Serum liver function measures including **g**, aspartate transaminase (AST; n=6 mice per condition); **h**, alanine transaminase (ALT; n=6 mice per condition); **i,** gamma-glutamyl transferase (GGT; Veh: n=5; SC9: n=6); and **j**, bilirubin (Veh: n=5; SC9: n=6). **k-m**, Serum kidney and muscle toxicity measures including: **k,** creatinine (Veh: n=5 mice; SC9: n=6 mice); **l**, creatine kinase (n=4 mice per condition); and **m,** lactate dehydrogenase (LDH; Veh: n=3 mice; SC9: n=4 mice). Serum hematology measures including: **n**, white blood cell count (WBC; n=6 mice per condition); **o**, hemoglobin (Hgb; ; n=6 mice per condition); **p,** hematocrit (Hct; ; n=6 mice per condition); and **q,** platelet count (PLT; ; n=6 mice per condition). Error bars show mean with SEM. A t-test was used to compare measures with two groups and one-way ANOVA with Dunnett's multiple comparisons test (each group against Veh., two-tailed test) was used for multiple groups. P-value results indicated by stars (ns = p > .05, * = p < .05, ** = p < .01, and **** = p < .0001). Non-significant p-values < .1 are written above bar. Source data including all statistics (degrees of freedom, p values, effect sizes, and confidence intervals) are provided in the Source Data file.

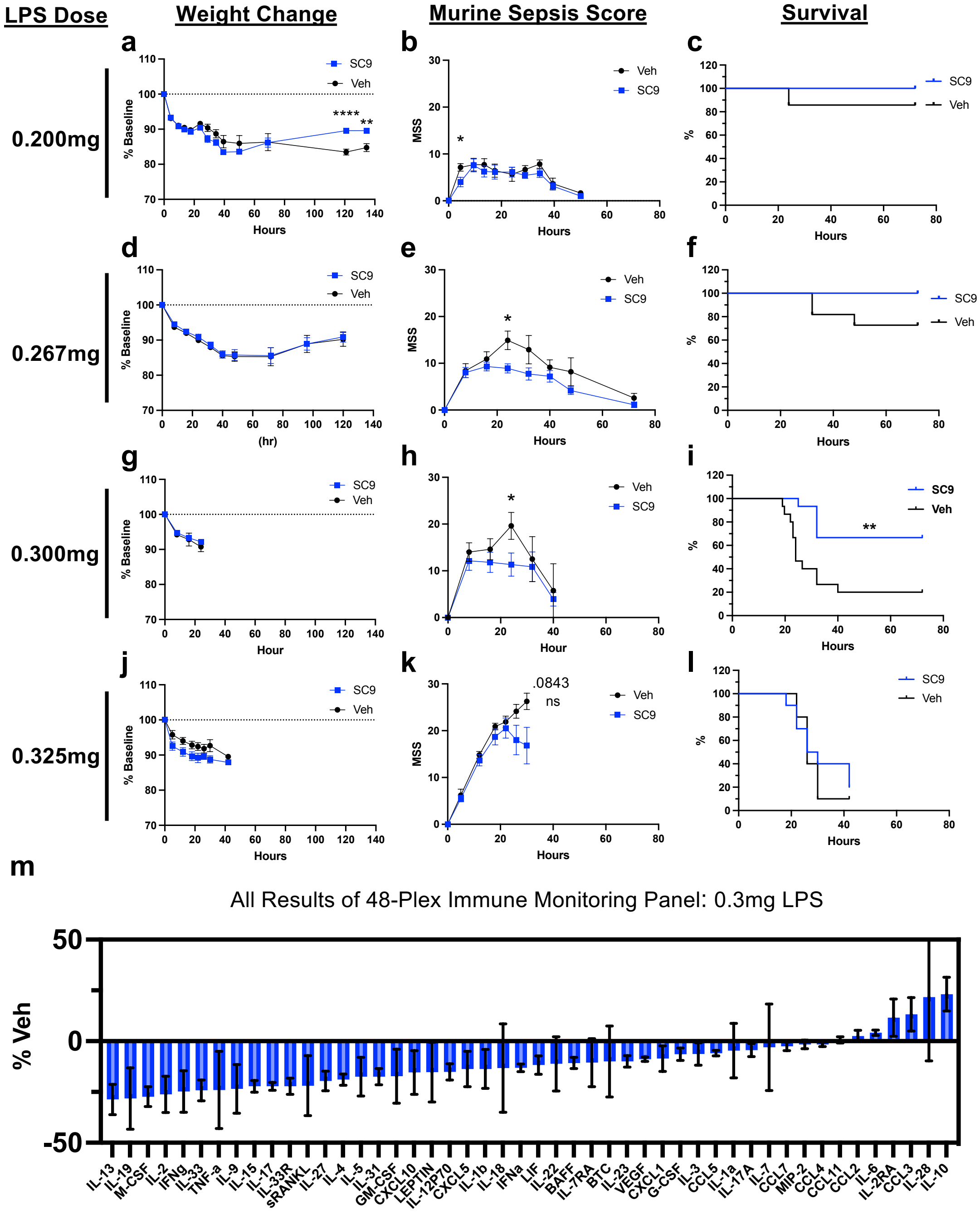

Supplementary Fig. 10| LPS-induced mouse endotoxemia model and the benefit of SC9 (10mg/kg). Effect on mouse body weight (left), cumulative murine sepsis score (center), and survival curve (right) in four experiments with the following LPS doses: 0.2mg **(a-c)** with n=7 mice per condition, 0.267mg **(d-f)** with n=10 mice per condition, 0.3mg **(g-i)** with n=15 mice per condition, and 0.325mg **(j-l)** with n=10 mice per condition. **m,** All of the results from the ProcartaPlex panel after SC9 treatment and LPS co-treatment showing average fold-change relative to Veh-treated mice (Veh: n=3 mice; SC9: n=4 mice). Error bars show mean with SEM. Statistical tests are a two-tailed t-test of SC9 vs. Veh. P-value results indicated by stars (ns = p > .05, * = p < .05, **). Non-significant p-values < .1 are written above bar. Source data including all statistics (degrees of freedom, p values, effect sizes, and confidence intervals) are provided in the Source Data file.

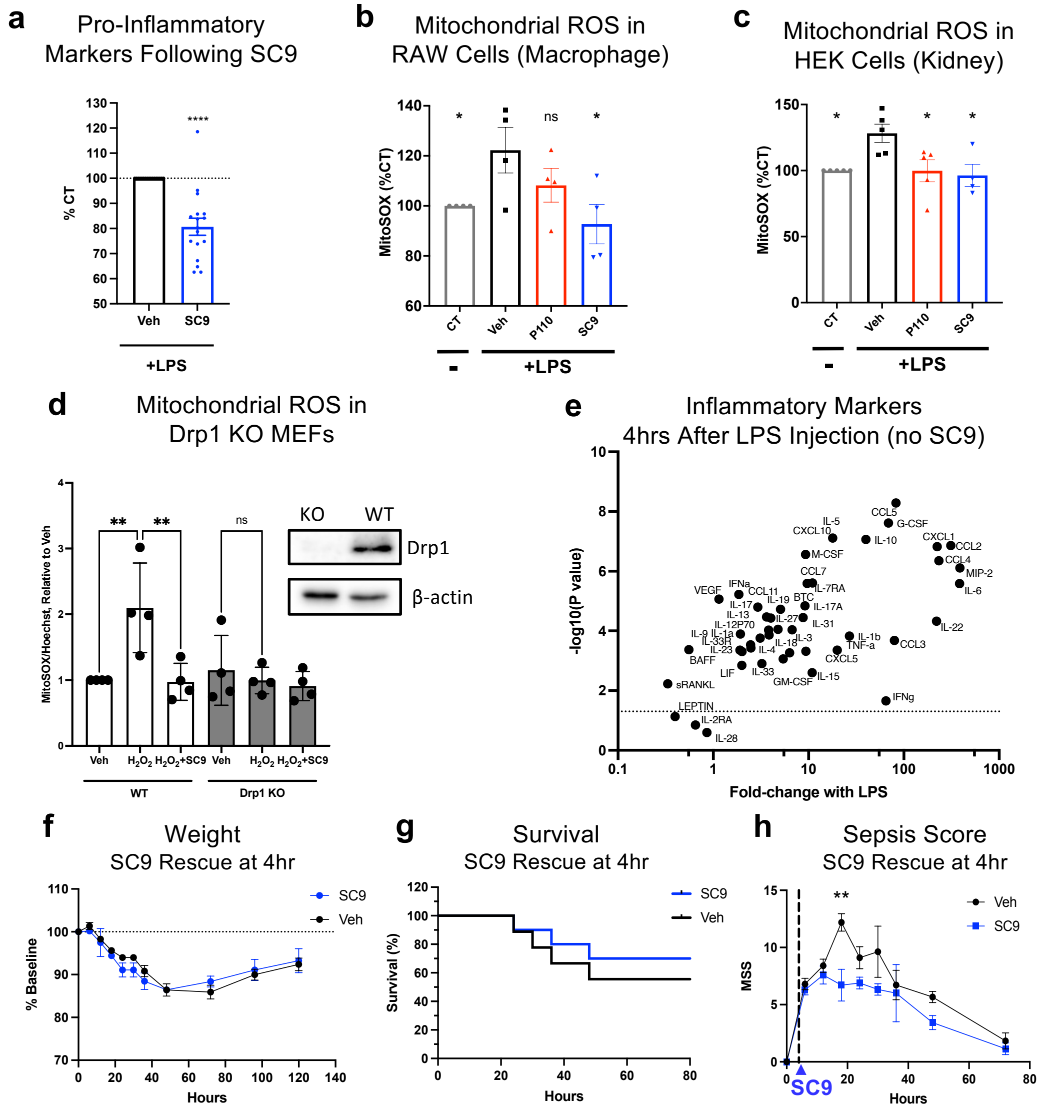

Supplementary Fig. 11| Additional SC9 validation data. a, Average values of each inflammatory analyte (IL-1a, I IL-1b, IL-2, IL-9, IL-6, L-15, IL-17, IL-17A, IL-18, IL-23, IL-27, IL-31, BAFF, IFN-α, IFN-γ, sRANKL, TNF-α) from the 48-Plex mouse ProcartaPlex panel normalized to vehicle mean. Plasma taken 4hrs after 0.3mg LPS treatment (Veh: n=3 mice; SC9: n=4 mice). **b,c**, Normalized MitoSOX fluorescence signal (MitoSOX/Hoechst) after 6hrs with LPS (2µg/mL) and treatment (2µM P110 and 0.5µM SC9) in **(b)** RAW 264.7 (mouse monocyte/macrophage-like cells) and **(c)** HEK293 (human embryonic kidney cells). Each point is the mean of 4-5 technical replicates from 4 independent experiments. **d,** Normalized MitoSOX fluorescence signal (MitoSOX/Hoechst) after 3hrs with peroxide (200µM) and treatment (1µM SC9) in Wild-type or Drp1-knockout mouse embryonic fibroblasts (MEF). Image at top right is a western blot of cell lysate (n=3 lysates). Each point is the mean of 4 technical replicates from 4 independent experiments. **e**, Values of all plasma analytes from the 48-Plex mouse ProcartaPlex panel normalized to vehicle mean. Plasma taken 4hrs after 0.3mg LPS treatment (CT: n=3 mice, LPS: n=3 mice). **f-h,** Effect on mouse body weight (**f**), survival (**g**), and sepsis score (**h**) in SC9 rescue experiment, delivered (20mg/kg) 4hrs and 8 hrs after injection of 0.3mg LPS (n=10 mice per condition). All error bars show mean with SEM. A two-tailed t-test was used to compare measures with two groups and one-way ANOVA with Dunnett's multiple comparisons test (each group against Veh., two-tailed test) was used for multiple groups. P-value results indicated by stars (ns = p > .05, * = p < .05, and ** = p < .01). Non-significant p-values < .1 are written above bar. MitoSOX intensity measurements were made in a 96-well florescence spectrometer. Source data including all statistics (degrees of freedom, p values, effect sizes, and confidence intervals) are provided in the Source Data file.
