# Supplementary material for: Targeting an allosteric site in dynamin-related protein 1 to inhibit Fis1-mediated mitochondrial dysfunction
Source: Nat Commun. 2023 Jul 19;14:4356. doi: 10.1038/s41467-023-40043-0 (PMC10356917; doi:10.1038/s41467-023-40043-0)
Supplement: Supplementary file 3 — Reporting Summary [file 41467_2023_40043_MOESM3_ESM.pdf]

nature portfolio

Corresponding author(s): Daria Mochly-Rosen

Last updated by author(s): Jun 26, 2023

Reporting Summary

Nature Portfolio wishes to improve the reproducibility of the work that we publish. This form provides structure for consistency and transparency in reporting. For further information on Nature Portfolio policies, see our Editorial Policies and the Editorial Policy Checklist.

Statistics

For all statistical analyses, confirm that the following items are present in the figure legend, table legend, main text, or Methods section.

n/a	Confirmed	
☐	☒	The exact sample size (*n*) for each experimental group/condition, given as a discrete number and unit of measurement
☐	☒	A statement on whether measurements were taken from distinct samples or whether the same sample was measured repeatedly
☐	☒	The statistical test(s) used AND whether they are one- or two-sided<br>*Only common tests should be described solely by name; describe more complex techniques in the Methods section.*
☐	☒	A description of all covariates tested
☐	☒	A description of any assumptions or corrections, such as tests of normality and adjustment for multiple comparisons
☐	☒	A full description of the statistical parameters including central tendency (e.g. means) or other basic estimates (e.g. regression coefficient) AND variation (e.g. standard deviation) or associated estimates of uncertainty (e.g. confidence intervals)
☐	☒	For null hypothesis testing, the test statistic (e.g. *F*, *t*, *r*) with confidence intervals, effect sizes, degrees of freedom and *P* value noted<br>*Give P values as exact values whenever suitable.*
☒	☐	For Bayesian analysis, information on the choice of priors and Markov chain Monte Carlo settings
☒	☐	For hierarchical and complex designs, identification of the appropriate level for tests and full reporting of outcomes
☒	☐	Estimates of effect sizes (e.g. Cohen's *d*, Pearson's *r*), indicating how they were calculated

Our web collection on statistics for biologists contains articles on many of the points above.

Software and code

Policy information about availability of computer code

Data collection	Microscope data were collected and processed with Keyence BZ-X Analyzer software (Version 1.3.0.3).
Data analysis	Imaging data were analyzed with an ImageJ macro (version 1.53t) which subtracted background, created binary images, and calculated the particle properties. Full focus images were generated using Keyence BZ-X Viewer (Version 1.3.0.5). This macro can be made available. Data analysis was done in Prism 9.3.1. Essential Site Scanning Analysis (ESSA) was done with publicly available script in Python 3.

For manuscripts utilizing custom algorithms or software that are central to the research but not yet described in published literature, software must be made available to editors and reviewers. We strongly encourage code deposition in a community repository (e.g. GitHub). See the Nature Portfolio guidelines for submitting code & software for further information.

Data

Policy information about availability of data

All manuscripts must include a data availability statement. This statement should provide the following information, where applicable:

- Accession codes, unique identifiers, or web links for publicly available datasets
- A description of any restrictions on data availability
- For clinical datasets or third party data, please ensure that the statement adheres to our policy

All the data generated in this study are provided in the Supplementary Information/Source Data file. Previously published crystal structures used in this study are available in the Protein Data Bank (PDB) under accession codes 4BEJ [https://doi.org/10.2210/pdb4BEJ/pdb], 3W6P [https://doi.org/10.2210/pdb3W6P/pdb] and

3W6O [https://doi.org/10.2210/pdb3W6O/pdb].The Any data raw and/or analyzed that support the findings of this study are available from the corresponding author upon reasonable request.

Human research participants

Policy information about studies involving human research participants and Sex and Gender in Research.

Reporting on sex and gender	N/A
Population characteristics	N/A
Recruitment	N/A
Ethics oversight	N/A

Note that full information on the approval of the study protocol must also be provided in the manuscript.

Field-specific reporting

Please select the one below that is the best fit for your research. If you are not sure, read the appropriate sections before making your selection.

☒ Life sciences ☐ Behavioural & social sciences ☐ Ecological, evolutionary & environmental sciences

For a reference copy of the document with all sections, see nature.com/documents/nr-reporting-summary-flat.pdf

Life sciences study design

All studies must disclose on these points even when the disclosure is negative.

Sample size	Minimum sample sizes were determined with a 80% power calculation using estimated effect size and standard deviation. However, more data was collected when possible to ensure statistical power.
Data exclusions	No data exclusions
Replication	2-3 independent studies were preformed for all experiments. No replication experiments were excluded.
Randomization	For recombinant Drp1 studies, samples were processed in parallel and reagents split from a well-mixed aliquot. Cell images were acquired at random locations within each well. Animal cages were randomly assigned to groups A and B. No other experiments required randomization.
Blinding	Manual cell segmentation for single cell quantification was done while blinded to experimental conditions. Animal cage group assignment, injections, sepsis scoring, and data analysis were done while blinded to conditions. Condition blindings were set and sealed in an envelope by independent researcher until the completion of the studies. All other assays did not required a subjective measurement and were not blinded.

Reporting for specific materials, systems and methods

We require information from authors about some types of materials, experimental systems and methods used in many studies. Here, indicate whether each material, system or method listed is relevant to your study. If you are not sure if a list item applies to your research, read the appropriate section before selecting a response.

Materials & experimental systems

n/a	Involved in the study	
☐	☒	Antibodies
☐	☒	Eukaryotic cell lines
☒	☐	Palaeontology and archaeology
☐	☒	Animals and other organisms
☒	☐	Clinical data
☒	☐	Dual use research of concern

Methods

n/a	Involved in the study	
☒	☐	ChIP-seq
☒	☐	Flow cytometry
☒	☐	MRI-based neuroimaging

Antibodies

Antibodies used	Mouse anti-Drp1 (611113; BD Biosciences; Lot#: 7130917; Clone 8/DLP1 (RUO)), rabbit anti-Fis1 (10956-1-AP; Proteintech), rabbit

Antibodies used	anti-MFF (17090-1-AP; Proteintech), goat anti-Rabbit IgG (H+L) DyLight 488 (35552; Invitrogen; Lot#: TK2666875; RRID AB_844398), goat anti-Mouse IgG (H+L) DyLight 650 (84545; Invitrogen; Lot#: VK309054; RRID AB_10942301), rabbit anti-Drp1 monoclonal antibody (Cell Signaling Technology; D6C7; #8570; Lot:5), mouse anti-B-Actin (Cell Signaling Technology; 8H10D10; #3700; Lot: 17), anti-Drp1 rabbit monoclonal antibody (Cell Signaling Technology; D8H5, #5391; Lot: 3), anti-a-Tubulin mouse monoclonal antibody (Cell Signaling Technology; DM1A; #3873; Lot: 16). HRP-linked anti-mouse IgG (Cytiva life Sciences; NA931, Lot#17317435). HRP-linked anti-rabbit IgG (Cytiva life Sciences; NA934, Lot#17187089).
Validation	KO/KD verified by Proteintech: 10956-1-AP and 17090-1-AP (see manufacturers website: https://www.ptglab.com). Cell Signaling Technology 8570 and BD Bioscience 611113 validated in-house with Drp1-KO MEFs. All other anti-bodies from Cell Signaling Technology and Cytiva are listed as passing "rigorous quality control" however the manufacturer does not list any additional validation data and no further validation was conducted in-house.

Eukaryotic cell lines

Policy information about cell lines and Sex and Gender in Research

Cell line source(s)	H9c2(2-1) strain BD1X (ATCC: CRL-1446;), RAW 264.7 (ATCC: TIB-71), HEK-293 (ATCC: CRL-1573), and MEFs were from Dr. Hiromi Sesaki from John Hopkins University (J Cell Sci. 2013 Feb 1; 126(3): 789–802).
Authentication	ATCC confirmed the authenticity of their cell lines lots with STR profiling using multiplex PCR, karyotyping, and cell morphology. Reference provided above for MEFs.
Mycoplasma contamination	ATCC has tested their cell line lots for mycoplasma and all lots used were negative.
Commonly misidentified lines (See ICLAC register)	No commonly misidentified lines were used.

Animals and other research organisms

Policy information about studies involving animals; ARRIVE guidelines recommended for reporting animal research, and Sex and Gender in Research

Laboratory animals	Female BALB/cAnNCrl mice, 7 weeks old (Charles River; Strain Code 028); male and female C57Bl6/J mice (Chareles River; Strain Code: 632)
Wild animals	N/A
Reporting on sex	Female animals were used in the LPS study however the response to LPS in male animals has been shown to be comparable. SC9 safety testing was done in both male and female mice.
Field-collected samples	N/A
Ethics oversight	All animal experiments were carried out under the protocols (APLAC-33002) approved by the Institutional Animal Care and Use Committee of Stanford University.

Note that full information on the approval of the study protocol must also be provided in the manuscript.
